# Long noncoding RNA TUG1 regulates the progression of colorectal cancer through miR-542-3p/TRIB2 axis and Wnt/β-catenin pathway

**DOI:** 10.1186/s13000-021-01101-7

**Published:** 2021-05-24

**Authors:** Quanlin Liu, Wei Zhang, Linshan Luo, Keshun Han, Ruitao Liu, Shue Wei, Xiaoran Guo

**Affiliations:** 1 Department of Colorectal Surgery, Zhengzhou Anorectal Hospital, No. 51, Longhai East Road, 450004 Zhengzhou, China; 2 Department of Constipation, Zhengzhou Anorectal Hospital, Zhengzhou, China; 3 Department of Large Intestine, Zhengzhou Anorectal Hospital, Zhengzhou, China

**Keywords:** Colorectal cancer, TUG1, miR-542-3p, TRIB2, Wnt/β-catenin pathway

## Abstract

**Background:**

Colorectal cancer (CRC) is one of the third normal malignancy worldwide. Taurine-upregulated gene 1 (TUG1), a member of long noncoding RNAs (lncRNAs), has been reported to be involved in various cancers. However, the mechanism underlying TUG1 in the progression of CRC remains unclear.

**Methods:**

The expression of TUG1, microRNA-542-3p (miR-542-3p), and tribbles homolog 2 (TRIB2) in CRC tissues and cells (LoVo and HCT116) were detected by quantitative real-time PCR (qRT-PCR). Methyl thiazolyl tetrazolium (MTT), transwell and flow cytometry assays were employed to evaluate the effects of TUG1 in CRC cells. The interaction between miR-542-3p and TUG1 or TRIB2 were verified by dual-luciferase reporter assay. A xenograft tumor model in nude mice was established to investigate the biological role of TUG1 in CRC *in vivo*.

**Results:**

TUG1 was increased in CRC tissues and cells (LoVo and HCT116) in contrast with adjacent normal tissues and normal intestinal mucous cells (CCC-HIE-2). Downregulation of TUG1 or TRIB2 suppressed the proliferation, migration, invasion, and induced apoptosis in CRC cells. And knockdown of TUG1 repressed tumor growth *in vivo*. Besides, overexpression of TRIB2 reversed the effects of TUG1 depletion on the progression of CRC. Meanwhile, TUG1 interacted with miR-542-3p and TRIB2 was a target of miR-542-3p. Furthermore, miR-542-3p knockdown or TRIB2 overexpression partly reversed the suppression effect of TUG1 depletion on the Wnt/β-catenin pathway.

**Conclusions:**

TUG1 served as a tumor promoter, impeded the progression of CRC by miR-542-3p/TRIB2 axis to inactivate of Wnt/β-catenin pathway, which providing a novel target for CRC treatment.

**Supplementary Information:**

The online version contains supplementary material available at 10.1186/s13000-021-01101-7.

## Background

Colorectal cancer (CRC) is a dominant cause of cancer death in US [1], and the five-year survival rate changes from about 90 % in patients with early tumor stage (I) to approximately 10 % in patients with advanced tumor stage (IV) [2]. Therefore, it is imperative to find novel therapy methods and molecular targets for CRC treatment.

Long non-coding RNAs (lncRNAs) are a type of RNA molecules ( more than 200 nucleotides ) and lose the ability of encoding proteins [3]. LncRNAs are reported to participate in various cancers [4, 5], including CRC [6, 7]. Previous reports have demonstrated that lncRNA taurine upregulated gene 1 (TUG1) is dysregulated in hepatocellular carcinoma [8], glioma [9] and prostate cancer [10]. Sun et al. found that TUG1 resulted in the low survival rate of CRC patients [11]. In fact, some studies have confirmed that TUG1 could boost CRC cell growth and metastasis through interacting with several target miRNAs [12, 13]. Yet, the precise mechanism of TUG1 in modulating the progression of CRC has not been fully addressed.

MicroRNAs (miRNAs) are short (about 22 nucleotides) and highly conserved noncoding RNAs, which regulate gene expression by guiding Argonaute proteins to target sites in the 3’-untranslated region (3’UTR) of messenger RNA (mRNA) [14]. Recent advances in cancers have profoundly deepened our comprehension of the important role of miRNAs in the progression of human cancers [15, 16]. Growing research has confirmed that miR-542-3p regulated the development and progression of CRC [17, 18], but the association between TUG1 and miR-542-3p in CRC is still unknown.

Tribbles homolog 2 (TRIB2) is an atypical protein kinase and it is considered as a potential allosteric drug target [19]. A previous study showed that TRIB2 conferred resistance to various chemotherapeutics [20]. Hou et al. observed that TRIB2 was highly expressed in CRC tissues and it could inhibit cellular senescence of CRC [21]. Hence, TRIB2 may be an attractive drug target for CRC and new regulators regulating the expression of TRIB2 require to be determined.

In this research, we found that TUG1 and TRIB2 were increased in CRC tissues and cells. Moreover, the knockdown of TUG1 inhibited proliferation, migration, invasion, and promoted apoptosis in CRC cells. Furthermore, bioinformatics analysis discovered that there were some binging sites between miR-542-3p and TUG1 or TRIB2. Therefore, we aim to validate the role of TUG1 in CRC progression, and to illuminate whether the involvement of TUG1 in CRC function was mediated by miR-542-3p/ TRIB2 axis.

## Materials and methods

### Samples and cell culture

 The research was conducted according to the guidelines of Declaration of Helsinki and was approved by the Ethics Committee of Zhengzhou Anorectal Hospital. CRC tissues and adjacent normal tissues were acquired 50 CRC patients, who received radical excision at Zhengzhou Anorectal Hospital from December 2015 to January 2019. The samples were frozen in liquid nitrogen immediately for further analysis. All patients involved in this study signed the written informed consents. None of the patients received chemotherapy or radiotherapy before surgery. And the clinicopathological features of the patients were shown in Table 1.

**Table 1:** Correlation between clinicopathological characteristics and TUG1 expression level in colorectal cancer

Characteristics	n	TUG1		*P*
		High	Low	
Gender				
Male	28	16	12	0.254
Female	22	9	13	
Age(years)				
≥50	25	14	11	0.396
<50	25	11	14	
TNM stage				
I + II	26	8	18	0.005*
III + IV	24	17	7	
Histological differentiation				
Well	23	7	16	0.011*
Poorly+Moderately	27	18	9	
Lymph node Metastasis				
No	26	9	17	0.024*
Yes	24	16	8	

Human normal intestinal mucous cell line (CCC-HIE-2) and CRC cell lines (LoVo and HCT116) were purchased from the Chinese Academy of Medical Sciences (Beijing, China). All cells were cultured in Dulbecco’s modified Eagle’s (DMEM, Sigma, St Louis, MO, USA) medium containing 10 % fetal bovine serum (FBS) (Sigma) in a humidified incubator with 5 % CO_2_. Cells were passaged every 2 days.

### Cell transfection

Small interfering RNA (siRNA) against TUG1 (si-TUG1#1 or si-TUG1#2, final concentration, 20 nM) or TRIB2 (si-TRIB2#1 or si-TRIB2#2, final concentration, 20 nM), miR-542-3p mimic (miR-542-3p, final concentration, 15 nM) and miR-542-3p inhibitor (anti-miR-542-3p, final concentration, 25 nM), as well as their corresponding controls (si-NC, final concentration, 20 nM; miR-NC, final concentration, 15 nM; anti-miR-NC, final concentration, 25 nM), were obtained from GenePharma (Shanghai, China). TUG1 expression plasmid (pcDNA-TUG1, final concentration, 1.6 µg/mL), TRIB2 expression plasmid (pcDNA-TRIB2, final concentration, 1.6 µg/mL) and the matched control (pcDNA, final concentration, 1.6 µg/mL) were acquired from RiboBio (Guangzhou, China). Cells transfection was carried out using Lipofectamine 2000 reagent (Invitrogen, Carlsbad, CA, USA) following the manufacturer’s recommendation. After 48 h incubation, transfected cells were harvested and utilized for further experiments.

### RNA isolation and quantitative real‐time polymerase chain reaction (qRT-PCR)

Total RNA of samples was isolated by FastPure Cell/Tissue Total RNA Isolation Mini Kit (Vazyme, Nanjing, China) and then the RNA was reversely transcribed to complementary DNA (cDNA) by PrimeScript™ RT Master Mix kit (Takara, Dalian, China). The qRT-PCR was conducted by SYBR Green PCR Master Mix (Vazyme). The relative RNA expressions were calculated by 2^−ΔΔCt^ method, normalizing to Beta-actin (β-actin) and U6. Primers in this study: TUG1 (forward, 5’-CTGAAGAAAGGCAACATC-3’, reverse, 5’-GTAGGCTACTACAGGATTTG-3’); miR-542-3p (forward, 5’-TGTGACAGATTGATAACTGAAA-3’, reverse, 5’-GTGCAGGGTCCGAGGT-3’); TRIB2 (forward, 5’-ATGAACATACACAGGTCTACCCC-3’, reverse, 5’-GGGCTGAAACTCTGGCTGG-3’); β-actin (forward 5’-GGATTCCTATGTGGGCGACGA-3’, reverse, 5’-GCGTACAGGGATAGCACAGC-3’); U6 (forward, 5’-GCTTCGGCAGCACATATACTAAAAT-3’, reverse, 5’-CGCTTCACGAATTTGCGTGTCA-3’).

###  Western blot analysis

Proteins from samples were isolated using RIPA buffer (Vazyme). The protein concentration was checked by Detergent Compatible Bradford Protein Quantification Kit (Vazyme). Proteins were segregated by 10 % sodium dodecyl sulfate polyacrylamide gel electrophoresis (SDS-PAGE) and then transferred onto the polyvinylidene difluoride (PVDF) membranes (Beyotime, Shanghai, China) which were blocked with 5 % skimmed milk (Beyotime). Afterward, the membranes were washed by Phosphate-buffered saline (PBS) and then incubated with the primary antibodies: anti-TRIB2 (1:1500, ab84683,Abcam, Cambridge, United Kingdom), anti-β-catenin (1:3000, ab32572, Abcam), anti-c-Myc (1:2500, ab32072, Abcam), anti-E-cadherin (1:3000, ab40772, Abcam) or anti-β-actin (1:3000, ab8227, Abcam)overnight. After being rewashed, the membranes were incubated with the secondary antibody (1:3000, ab205718, Abcam) for 3 h. The membranes were analyzed by the ChemiDoc™ MP Imaging System (Bio-Rad, Richmond, CA, USA) after being treated with ECL kit (Beyotime).

### 3- (4, 5-Dimethylthiazol-2-yl)-2, 5-Diphenyltetrazolium bromide (MTT) assay

MTT assay was conducted to measure cell proliferation. Briefly, transfected cells (5 × 10^3^ cells/well) were plated into 96-well plate. 20 µL MTT solutions (5 mg/mL) (Sigma) was added to the well to incubate for 4 h. Afterwards, 200 µL dimethyl sulfoxide (Sigma) was added to the well after discarding medium. Optical density values were examined at 490 nm wavelength under the microplate reader (Bio-Rad).

### Flow cytometry assay

Annexin Apoptosis Detection Kit (Sigma) was employed to evaluate cell apoptosis following the given procedures. In brief, at 48 h after transfection, 1 × 10^6^ cells were harvested and resuspended in provided binding buffer and then 5 µL Annexin V-fluorescein isothiocyanate (Annexin V-FITC) and 5 µL propidium iodide (PI) were added to the buffer for incubating for 5 min in the dark. The stained cells were analyzed by the flow cytometry (Thermo Fisher Scientific, Rockford, IL, USA).

### Transwell assay

Transwell chamber pre-coated with Matrigel (Corning Life Sciences, Corning, NY, USA) or not was employed to check the capacity of cell invasion or migration, respectively. Transfected cells at a density of 1 × 10^5^ cells/well were plated into the upper chamber and the DMEM medium was placed in the lower chamber. After being treated with crystal violet (Solarbio, Beijing, China), migrated or invaded cells were analyzed under an inverted microscope (MultiskanEX, Lab Systems, Helsinki, Finland).

### Dual‐luciferase reporter assay

The potential binding sites of miR-542-3p and TUG1 or TRIB2 were predicted by starBase [22]. The sequences of wild type TUG1 (WT-TUG1: 5’-TGGCTCTGCTGACCCTGTCACT-3’), mutant TUG1 (MUT-TUG1: 5’-TGGCTCTGCCGCCCACACCGTT-3’), wild type TRIB2 (TRIB2 3’UTR-WT) and mutant TRIB2 (TRIB2 3’UTR-MUT) harboring the putative target sites of miR-542-3p were cloned and inserted into the pGL3 vectors (Promega, Madison, WI, USA). Then, the vectors with miR-542-3p or miR-542-3p + pcDNA-TUG1, as well as corresponding controls, were co-transfected into LoVo and HCT116 cells using Lipofectamine 2000 (Invitrogen). At 48 h after transfection, the Dual-Glo® Luciferase Assay System kit (Promega) was used to detect the luciferase activity. Renilla luciferase activities were used as the internal control for the normalization of firefly luciferase activity.

### Xenograft mouse model

The short hairpin RNA against TUG1 (named as sh-TUG1: sense sequence, 5’-GCAGTAATTGGAGTGGATACG-3’, and antisense sequence, 5’-CGTATCCACTCCAATTACTGC-3’) and the negative control (named as sh-NC: sense sequence, 5’-GTTGAGAGACCGTTGAGTAGA-3’, and antisense sequence, 5’-TCTACTCAACGGTCTCTCAAC-3’) were constructed by BioSETTIA (San Diego, CA, USA). After the CRC cells were transfected with sh-NC (final concentration, 2 µg/mL) or sh-TUG1 (final concentration, 2 µg/mL) for 48 h, the stable cells were selected using 2 µg/mL for 7 weeks.

Six-week-old nude mice (*n* = 50) were obtained from the Shanghai Experimental Animal Center (Shanghai, China). Subsequently, these mice were fed in an SPF environment, with 12 h light/dark cycles, and constant and suitable temperature (25˚C) and humidity (60 %). The mice were randomly divided into two groups (experiment I and experiment II). All of the flank of the nude mice were subcutaneously injected with CRC cells stable transfected with sh-TUG1 or sh-NC to establish the xenograft mouse model.

Experiment I: A total of 10 mice were used for this research (5 mice for sh-NC group and 5 mice for sh-TUG1 group). After injection for 7 days, the weight of nude mice and tumor volume were calculated every 5 days for 27 days. Tumor volume was calculated according to the formula: tumor volume = 0.5 × length × width^2^. 27 days upon injection, the mice were euthanized, and xenograft tumor tissues were removed, weighed and stored in liquid nitrogen.

Experiment II: The mice in sh-TUG1 (*n* = 20) or sh-NC (*n* = 20) were used for research. At 7 days, 12 days, 17 days, 22 days and 27 days upon injection, 4 mice were euthanized, and xenograft tumor tissues were removed and weighed. Then the expression of TUG1, miR-542-3p and TRIB2 in xenograft tumor tissues were investigated by qRT-PCR.

### Statistical analysis

Experimental data were presented by mean ± standard deviation (SD). Two independent groups were compared by Student’s *t*-test. For more than two groups, the one-way analysis of variance (ANOVA) was used to assess the difference. Each experiment was conducted at least three times independently. *P* < 0.05 represented statistical significance.

## Results

### The expression levels of TUG1 and TRIB2 were significantly upregulated in CRC tissues

**Fig. 1:**
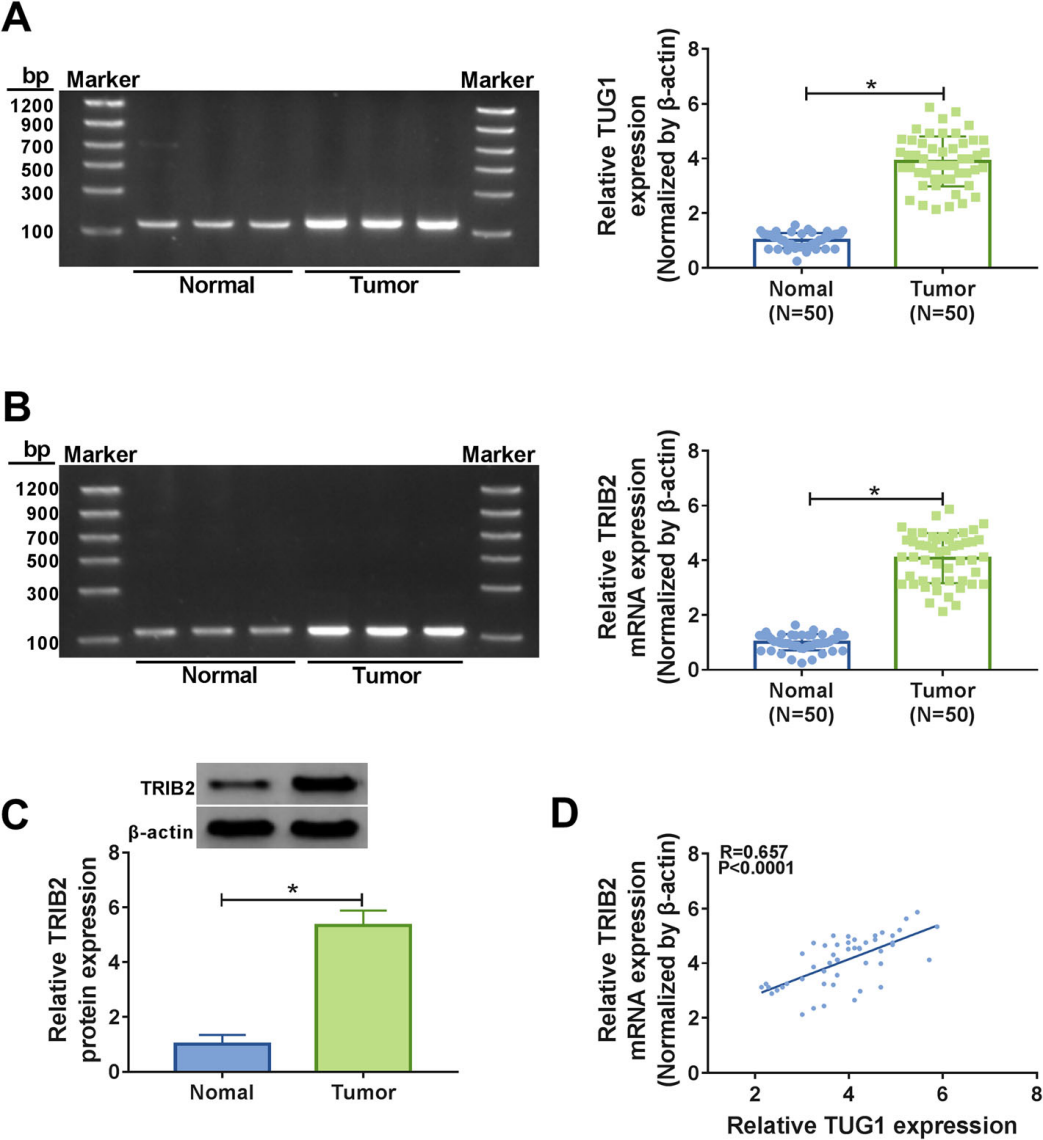
TUG1 and TRIB2 were upregulated in CRC tissues. **a, b** The expression levels of TUG1 and TRIB2 in normal tissues and tumor tissues were detected by qRT-PCR. And the specificity of amplification of each primer pair was verified by agarose gel visualization. **c** The protein level of TRIB2 in normal tissues and tumor tissues was checked by western blot. **d** The correlation between TUG1 and TRIB2 in tumor tissues was determined using Pearson’s correlation coefficient. *P < 0.05

To investigate the roles of TUG1 and TRIB2, we first detected their expression levels in CRC tissues. The data showed that TUG1 was conspicuously upregulated in CRC tissues compared with corresponding normal tissues (Fig. 1a). And we also investigated the association between TUG1 expression and the clinicopathological features of CRC patients. CRC patients were divided into high TUG1 expression group (*n* = 25) and low TUG1 expression group (*n* = 25). As displayed in Table 1, elevated expression of TUG1 exhibited a significant correlation with the TNM stage (*P* = 0.005), Histological differentiation (*P* = 0.011) and Lymph node Metastasis (*P* = 0.024), whereas it has no connection with the age and gender of CRC patients. These results indicating the close association between TUG1 and the prognosis of CRC. Furthermore, the mRNA and protein levels of TRIB2 were also significantly elevated in CRC tissues (Fig. 1b c). Moreover, correlation analysis indicated that the expression of TRIB2 was positively associated with TUG1 in CRC tissues (Fig. 1d).

### Knockdown of TUG1 inhibited proliferation, migration, invasion and induced apoptosis of CRC cells

**Fig. 2:**
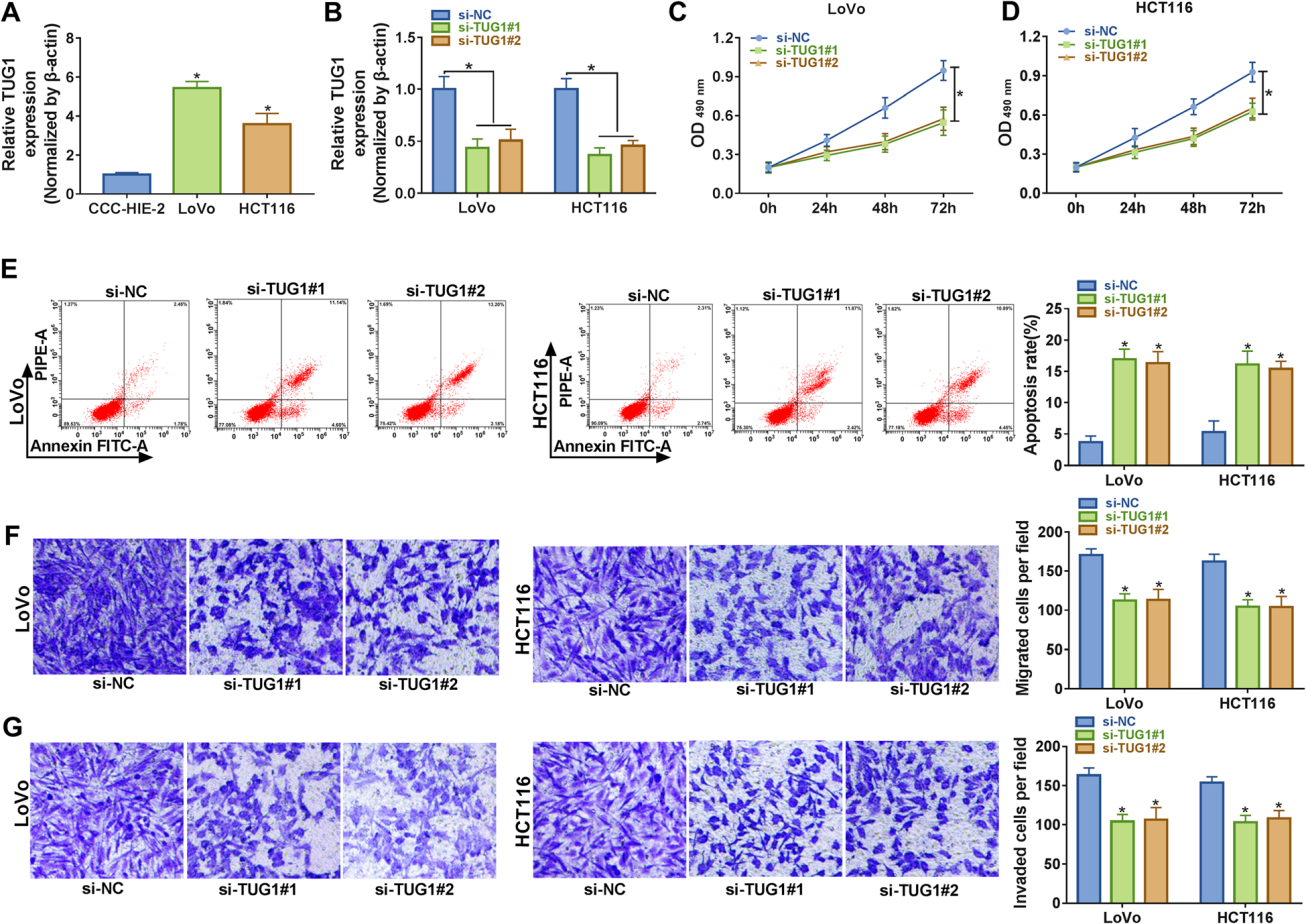
Downregulation of TUG1 inhibited proliferation, migration, invasion, and induced apoptosis of CRC cells. **a** The expression of TUG1 in CRC cell lines (LoVo and HCT116) and human normal intestinal mucous cell line (CCC-HIE-2) was evaluated by qRT-PCR. **b** The level of TUG1 in CRC cells transfected with si-NC or si-TUG1 was determined by qRT-PCR. **c, d** MTT assay was used to detect cell viability of LoVo and HCT116 cells transfected with si-NC or si-TUG1 at different time points. **e** Flow cytometry was used to detect apoptosis of transfected CRC cells at 48 h after transfection. **f, g** Transwell migration and invasion assays were performed, and the cell numbers of migration or invasion were calculated. *P < 0.05

To further explore the function of TUG1 in CRC, we checked its expression in CRC cell lines (LoVo and HCT116) and normal intestinal mucous cell line (CCC-HIE-2). The data indicated that TUG1 was strikingly upregulated in CRC cells (Fig. 2a). To study the possible role of TUG1 in CRC cell proliferation, apoptosis migration and invasion, CRC cells were transfected with si-TUG1 (si-TUG1#1 or si-TUG1#2) or si-NC, and the knockdown efficiency was tested by qRT-PCR (Fig. 2b). MTT assay showed that the proliferation of LoVo and HCT116 cells decreased significantly at 48 and 72 h upon si-TUG1#1 or si-TUG1#2 transfection compared with the si-NC group (Fig. 2c and d). Flow cytometry analysis revealed that the apoptosis rates of CRC cells transfected with TUG1 knockdown were notably increased (Fig. 2e). Besides, transwell assay elucidated that TUG1 depletion inhibited migration and invasion of CRC cells (Fig. 2f and g). Together, these results demonstrated that TUG1 silencing repressed proliferation, migration, invasion and promoted apoptosis of CRC cells *in vitro*.

### Downregulation of TRIB2 suppressed proliferation, migration, invasion and promoted apoptosis of CRC cells

**Fig. 3:**
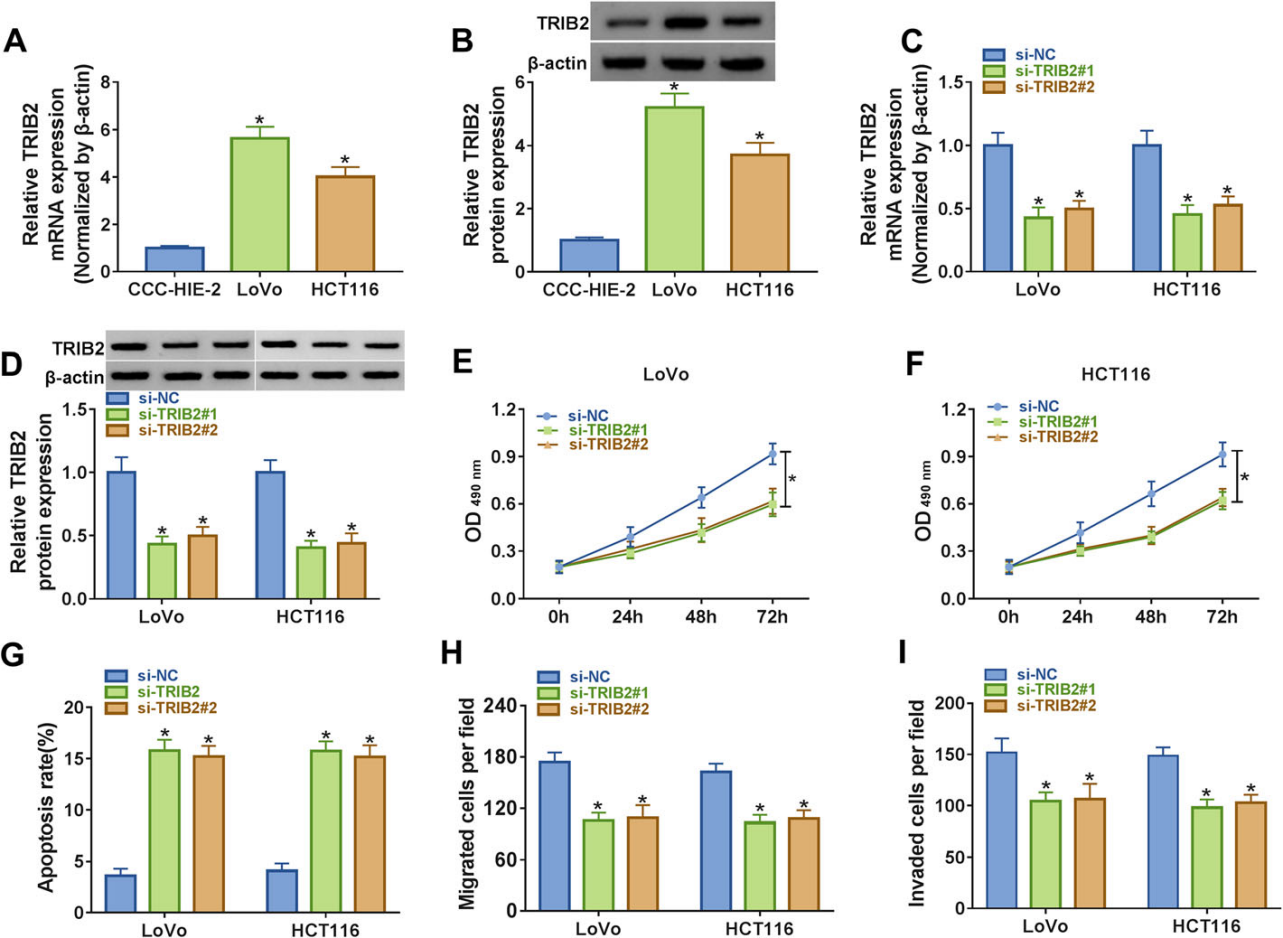
Knockdown of TRIB2 inhibited proliferation, migration, invasion, and induced apoptosis of CRC cells. **a, b** The mRNA and protein levels of TUG1 in CRC cells and normal cells were measured by qRT-PCR and western blot, respectively. **c, d** The mRNA and protein levels of TUG1 in CRC cells transfected with si-NC or si-TRIB2 were checked by qRT-PCR and western blot, respectively. **e, f** Proliferation of transfected CRC cells was assessed using MTT assay at different time points. **g** Flow cytometry was employed to check apoptosis of transfected CRC cells. **h, i** The abilities of migration and invasion of transfected CRC cells were evaluated by transwell assay. *P < 0.05

To study the possible function of TRIB2 in CRC, the mRNA and protein levels of TRIB2 in CRC cells were evaluated by qRT-PCR and western blot, respectively. The data showed that TRIB2 was dramatically increased in CRC cells compared with normal cells (Fig. 3a and b). Subsequently, the expression of TRIB2 in CRC cells transfected with si-TRIB2 (si-TRIB2#1 or si-TRIB2#2) or si-NC was detected, and the results indicated that the mRNA and protein levels of TRIB2 were significantly declined in cells with si-TRIB2#1 or si-TRIB2#2 transfection (Fig. 3c and d). MTT assay manifested that knockdown of TRIB2 inhibited proliferation of LoVo and HCT116 cells (Fig. 3e f). Moreover, the apoptosis rates of CRC cells were significantly elevated (Fig. 3g). Further analysis indicated that downregulation of TRIB2 weakened the capacities of migration and invasion of CRC cells (Fig. 3h and i). Collectively, these results suggested that downregulation of TRIB2 inhibited proliferation, migration, invasion and induced apoptosis of CRC cells.

### Overexpression of TRIB2 reversed the effects of TUG1 silencing-mediated repression on proliferation, migration, invasion and promotion on apoptosis of CRC cells

**Fig. 4:**
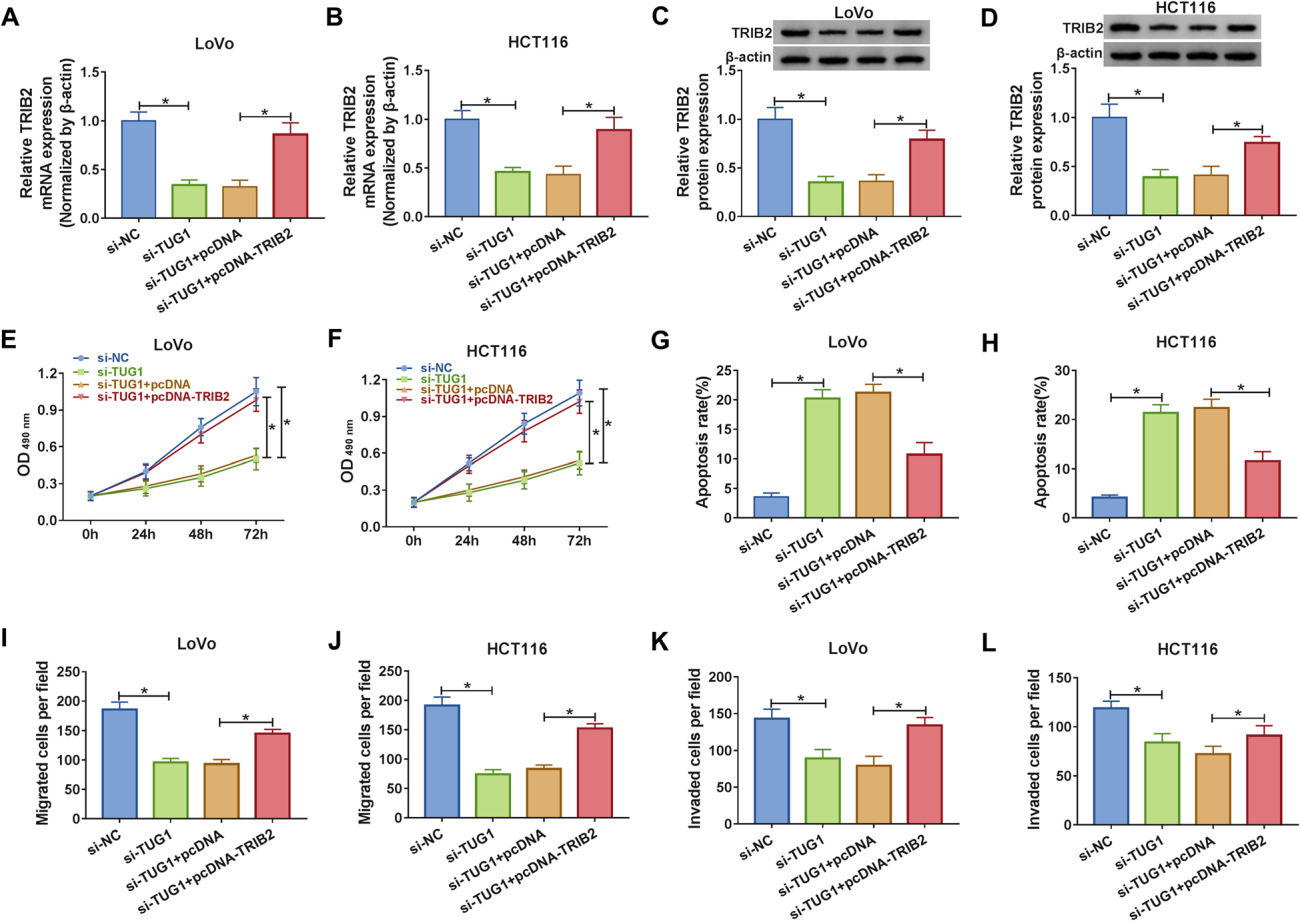
Overexpression of TRIB2 rescued the effects of TUG1 silencing-mediated on proliferation, migration, invasion and apoptosis of CRC cells. **a, b** The expression of TRIB2 in CRC cells transfected with si-TUG1 or si-TUG1 + pcDNA-TRIB2, as well as corresponding controls, was detected by qRT-PCR. **c, d** The protein level of TRIB2 in transfected CRC cells was measured by western blot. **e, f** Viability of transfected CRC cells was evaluated by MTT assay at different time points. **g, h** Apoptosis of transfected CRC cells was checked by flow cytometry. **i, j** Transwell chamber without Matrigel was hired to assess migration of transfected CRC cells. **k, l** Transwell chamber pre-coated with Matrigel was used to check the invasion of transfected CRC cells. *P < 0.05

From the results above, we knew that both TUG1 and TRIB2 were involved in the progression of CRC, which inspired our curiosity to explore the relationship between the two. The mRNA and protein expression levels of TRIB2 in CRC cells transfected with si-TUG1 or si-TUG1 + pc-DNA-TRIB2, as well as matched controls, were checked by qRT-PCR and western blot, respectively. The result showed that TUG1 silencing conspicuously downregulated the mRNA and protein expression of TRIB2, whereas the effects could be attenuated by the overexpression of TRIB2 (Fig. 4a-d). MTT assay indicated that overexpression of TRIB2 rescued the effect of TUG1 silencing-mediated inhibition on proliferation of CRC cells (Fig. 4e f). Moreover, upregulation of TRIB2 reversed the impact of TUG1-mediated promotion on apoptosis of CRC cells (Fig. 4g h). Transwell assay illustrated that knockdown of TUG1 suppressed migration and invasion of CRC cells, while overexpression of TRIB2 abolished these impacts (Fig. 4i l). To sum up, these results suggested that upregulation of TRIB2 could lessen the effects of TUG1 silencing-mediated on proliferation, apoptosis, migration and invasion of CRC cells *in vitro*.

### TUG1 modulated the expression TRIB2 by sponging miR-542-3p in CRC cells

**Fig. 5:**
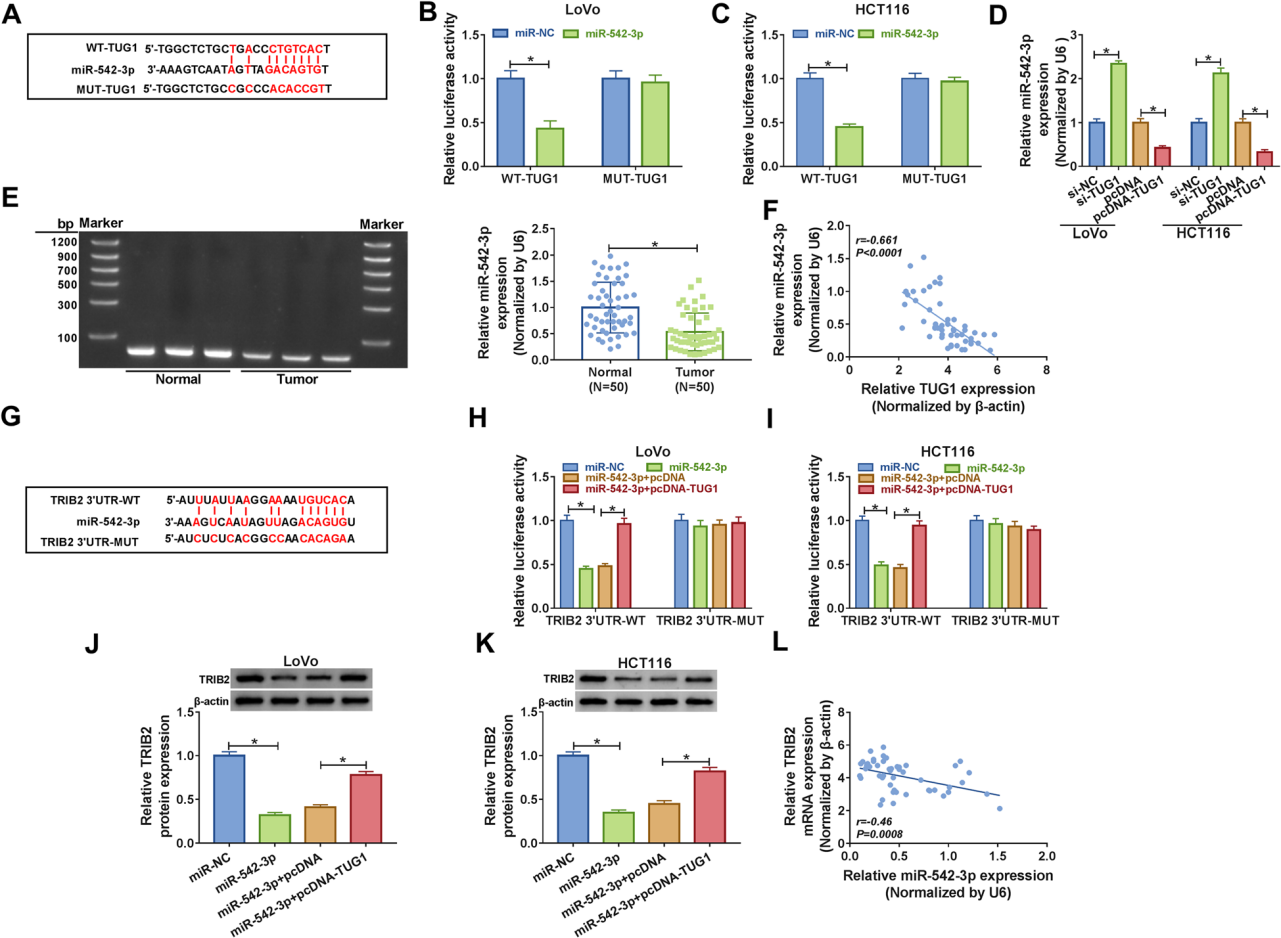
TUG1 regulated TRIB2 by interacting with miR-542-3p in CRC cells. **a** The putative binding sites between TUG1 and miR-542-3p were predicted by starBase. **b, c** The luciferase activity of CRC cells co-transfected with miR-542-3p and WT-TUG1 or MUT-TUG1 was checked. **d,** **e** The level of miR-542-3p in Lovo and HCT116 cells transfected with si-TUG1 or pcDNA-TUG1, as well as matched controls was determined by qRT-PCR. And the specificity of amplification of miR-542-3p primer pair was verified by agarose gel visualization. **f **Correlation between miR-542-3p expression and TUG1 expression was assessed by Pearson’s correlation coefficient. **g** The putative binding sites between miR-542-3p and TRIB2 were forecasted by starBase. **h, i** The Dual-luciferase reporter assay was performed to confirm the interaction between miR-542-3p and TRIB2. **j, k** The protein level of TRIB2 in CRC cells transfected with miR-542-3p or miR-542-3p + pcDNA-TUG1, as well as matched controls, was measured by western blot. **l** Pearson’s correlation coefficient for the relationship between miR-542-3p expression and TRIB2 expression. *P < 0.05

Bioinformatics analysis indicated that TUG1 was a target of miR-542-3p (Fig. 5a), and the dual-luciferase reporter assay was introduced to confirm the prediction. The data showed that miR-542-3p significantly diminished the luciferase activity of WT-TUG1 in LoVo and HCT116 cells, rather than MUT-TUG1 (Fig. 5b c). In addition, knockdown of TUG1 significantly upregulated the expression of miR-542-3p, whereas overexpression of TUG1 notably downregulated the expression of miR-542-3p (Fig. 5d and e). Besides, a negative correlation (*r* = -0.661, *P* < 0.0001) was found between miR-542-3p expression and TUG1 expression in 50 cases of CRC patients (Fig. 5f). Interestingly, miR-542-3p was predicted to bind to the 3’UTR of TRIB2 (Fig. 5g) and corresponding luciferase reporter plasmids (TRIB2 3’UTR-WT and TRIB2 3’UTR-MUT) were constructed to verify the interaction. The data indicated that miR-542-3p strongly decreased the luciferase activity of TRIB2 3’UTR-WT instead of TRIB2 3’UTR-MUT, but overexpression of TUG1 could increase the luciferase activity of TRIB2 3’UTR-WT (Fig. 5h and i). Then the protein level of TRIB2 in CRC cells transfected with miR-542-3p or miR-542-3p + pcDNA-TUG1, as well as matched controls, was detected by western blot. The result demonstrated that miR-542-3p led to significant suppression of TRIB2 expression in CRC cells, whereas the impact could be alleviated after the transfection of pcDNA-TUG1 (Fig. 5j and k). Besides, TRIB2 expression was negatively correlated (*r* = -0.46, *P* = 0.0008) with miR-542-3p expression in 50 cases of CRC patients (Fig. 5l). Taken together, these results suggested that TUG1 could regulate the expression of TRIB2 by interacting with miR-542-3p in CRC cells *in vitro*.

### TUG1 regulated the Wnt/β-catenin pathway by miR-542-3p/TRIB2 axis

**Fig. 6:**
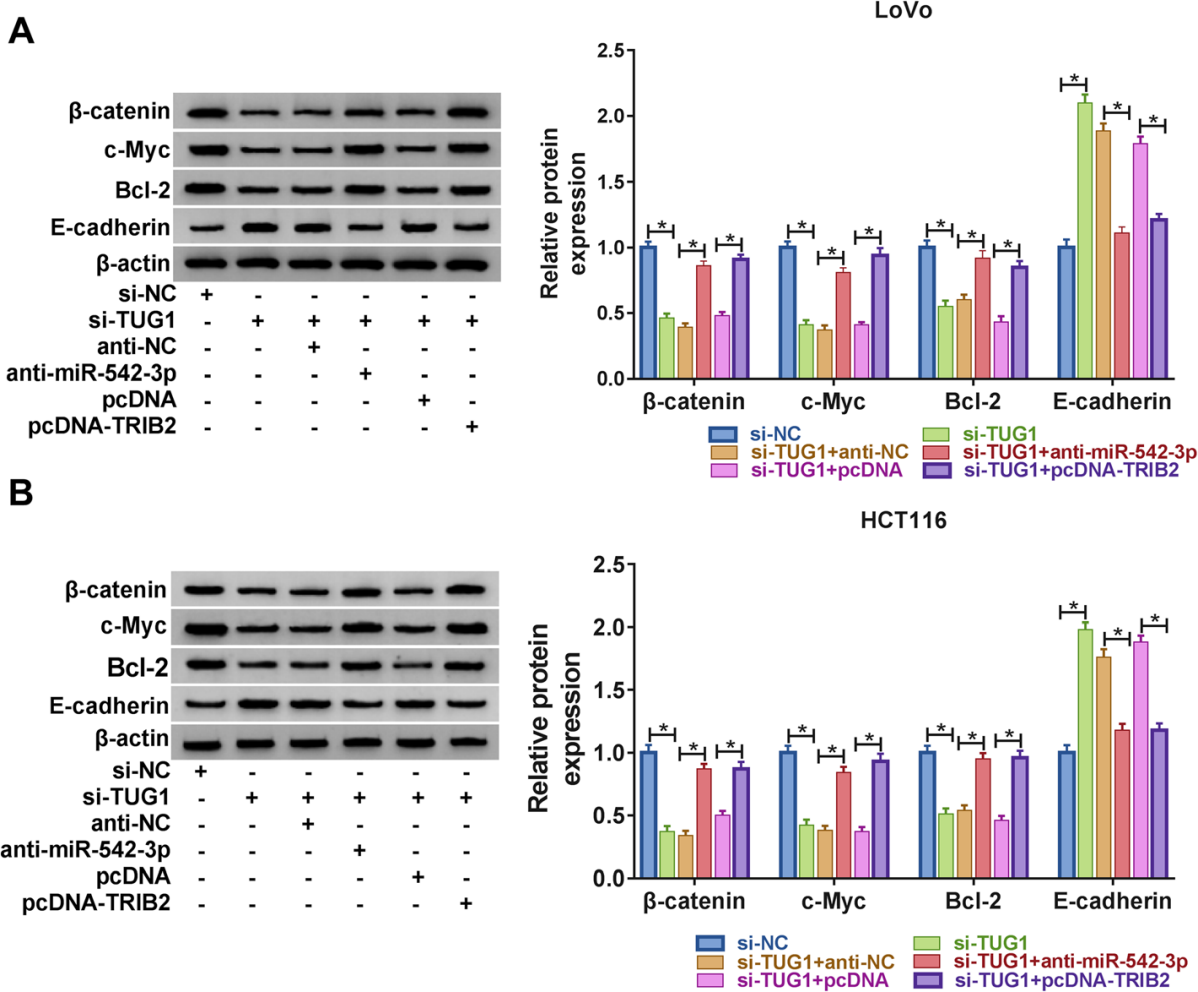
TUG1/miR-542-3p/TRIB2 axis regulated Wnt/β-catenin pathway. **a** The protein levels of β-catenin, c-Myc, Bcl-2 and E-cadherin in LoVo cells transfected with si-TUG1, si-TUG1 + anti-miR-542-3p or si-TUG1 + pcDNA-TRIB2, as well as their matched controls, were detected by western blot. **b** The protein levels of β-catenin, c-Myc, Bcl-2 and E-cadherin in transfected HCT116 cells were measured by western blot. *P < 0.05

TUG1 was reported to regulate Wnt/β-catenin signaling pathway to mediate the progression of multiple cancers, including CRC [23, 24]. Wnt/β-catenin pathway is among the regulatory pathways vital to the progression of diverse cancers, including cell apoptosis, cell cycle progression, apoptosis, migration and invasion [25, 26]. Thus, to further investigate whether TUG1 regulated Wnt/β-catenin pathway to mediate the progression of CRC, the expression levels of proteins (β-catenin, c-Myc, Bcl-2 and E-cadherin) associated with Wnt/β-catenin pathway in CRC cells transfected with si-TUG1, si-TUG1 + anti-miR-542-3p or si-TUG1 + pc-DNA-TRIB2, as well as the corresponding controls, were measured by western blot. The results disclosed that TUG1 silencing conspicuously reduced the expression of β-catenin, c-Myc, Bcl-2 and elevated the expression of E-cadherin in CRC cells, yet downregulation of miR-542-3p or overexpression of TRIB2 could reverse the effects (Fig. 6a and b). All in all, these results demonstrated that knockdown of TUG1 blocked the Wnt/β-catenin pathway by the regulation of TRIB2/miR-542-3p axis.

### Knockdown of TUG1 inhibited tumor growth *in vivo*

**Fig. 7:**
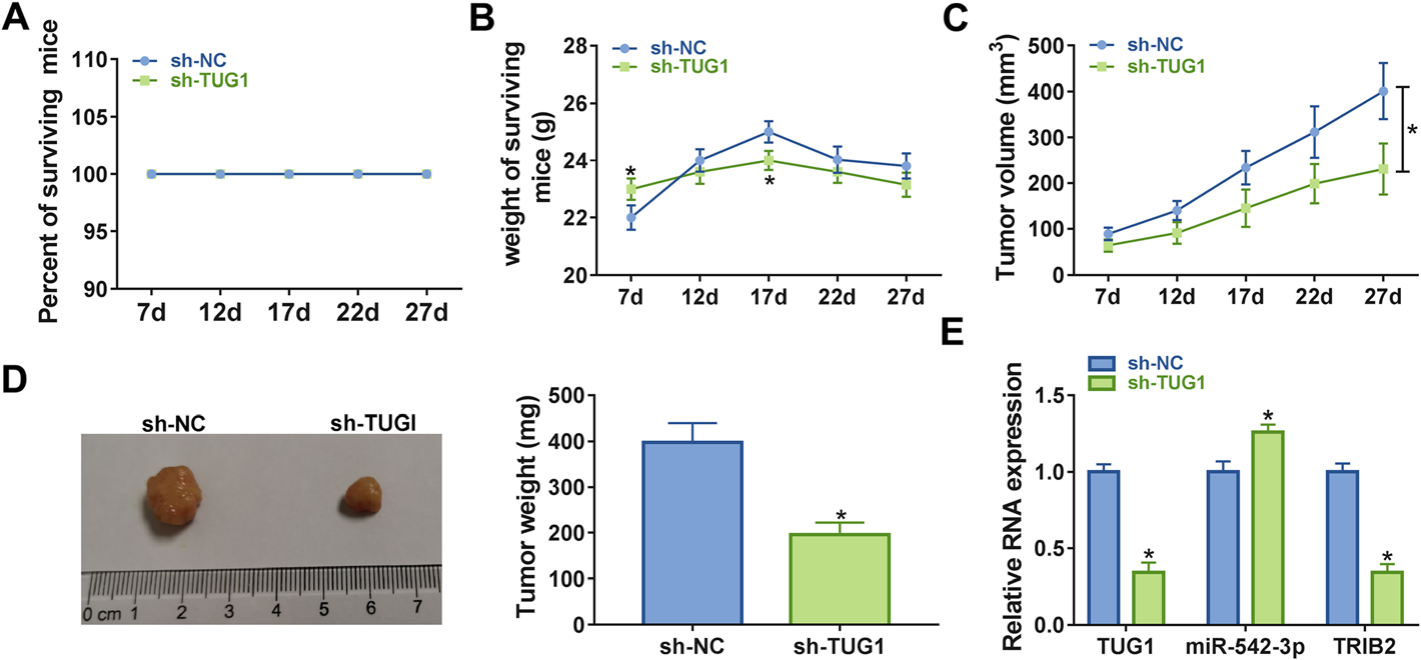
Knockdown of TUG1 inhibited the growth of tumor *in vivo*. **a** Survival rate of nude mice upon injection. **b** The weight of surviving mice upon injection for 7 days were weighed every 5 days for 27 days. **c** 7 days upon injection, tumor volume was measured every 5 days for 27 days. **d** Images and weight of the resected tumor was examined after the mice were killed. **e** The levels of TUG1, miR-542-3p and TRIB2 in CRC cells transfected with sh-NC or sh-TUG1 were measured by qRT-PCR. *P < 0.05

To confirm the function of TUG1 in the progression of CRC *in vivo*, we established the xenograft mouse model using the CRC cells transfected with sh-NC or sh-TUG1. The body weight and survival rates of the mice were recorded to evaluate the tolerance of nude mice to TUG1 knockdown. As shown in Fig. 7a and b, there were no mice dead before the end of the experiment, and the body weight of nude mice with TUG1 knockdown showed a slight lose in the day of 17 after injection. However, the body weight of nude mice with TUG1 knockdown was not significantly different than the sh-NC group at the end of the experiment, indicating that TUG1 knockdown is well tolerated in nude mice. Besides, TUG1 silencing decreased the tumor volume (Fig. 7c) and tumor weight (Fig. 7d) in contrast with sh-NC group. Then the expression levels of TUG1, miR-542-3p and TRIB2 in tumors were determined by qRT-PCR, and the results indicated that the expression of TUG1 and TRIB2 was apparently declined in sh-TUG1 group compared with sh-NC group, which was contrary to the expression of miR-542-3p (Fig. 7e). Furthermore, another experiment in nude mice indicated that relative expression of TUG1 and TRIB2 remain low while miR-542-3p expression was at high level in the sh-TUG1 group (Supplementary Fig. 1A-C). Thus, downregulation of TUG1 could suppress tumor growth by miR-542-3p/TRIB2 axis *in vivo*.

## Discussion

CRC threatens human health due to the low survival rate [2]. LncRNAs have been verified to be associated with the progression of CRC [27, 28]. Previous studies indicated that lncRNA TUG1 functioned in the development of CRC. Sun et al. found that TUG1 accelerated metastasis of CRC cells *in vivo* [29], and Zhai et al. confirmed that overexpression of TUG1 boosted proliferation and migration of CRC cells [30]. In our research, we measured the expression level of TUG1 and the result indicated that the expression of TUG1 was significantly elevated in CRC tissues and cells, which was in line with a previous report [29]. And the high expression of TUG1 was closely related to the poor outcomes of CRC patients. To study the function of TUG1 in the progression of CRC, the proliferation, apoptosis, migration and invasion of CRC cells transfected with si-TUG1 were checked. The data clarified that downregulation of TUG1 notably repressed proliferation, migration, invasion and induced apoptosis of CRC cells *in vitro*. These results demonstrated that TUG1 might acted as a tumor promoter in CRC progression.

 TRIB2, an atypical protein kinase, regulated the progression of cancers. Rishi et al. disclosed that TRIB2 was associated with acute myeloid leukemia [31]. Grandinetti et al. found that knockdown of TRIB2 inhibited proliferation of lung cancer cells and tumor growth *in vivo* [32]. Hill et al. reported that high expression of TRIB2 led to the poor clinical outcome of CRC [20], and Hou et al. confirmed that TRIB2 could repressed cellular senescence of CRC [21]. In this research, we observed that TRIB2 was conspicuously increased in CRC tissues and cells compared with corresponding controls, which was supported by Hou’s research [21]. Further analysis illustrated that downregulation of TRIB2 suppressed proliferation, migration, invasion and accelerated apoptosis of CRC cells. These results disclosed that TRIB2 participates in the regulation of CRC progression.

**Fig. 8:**
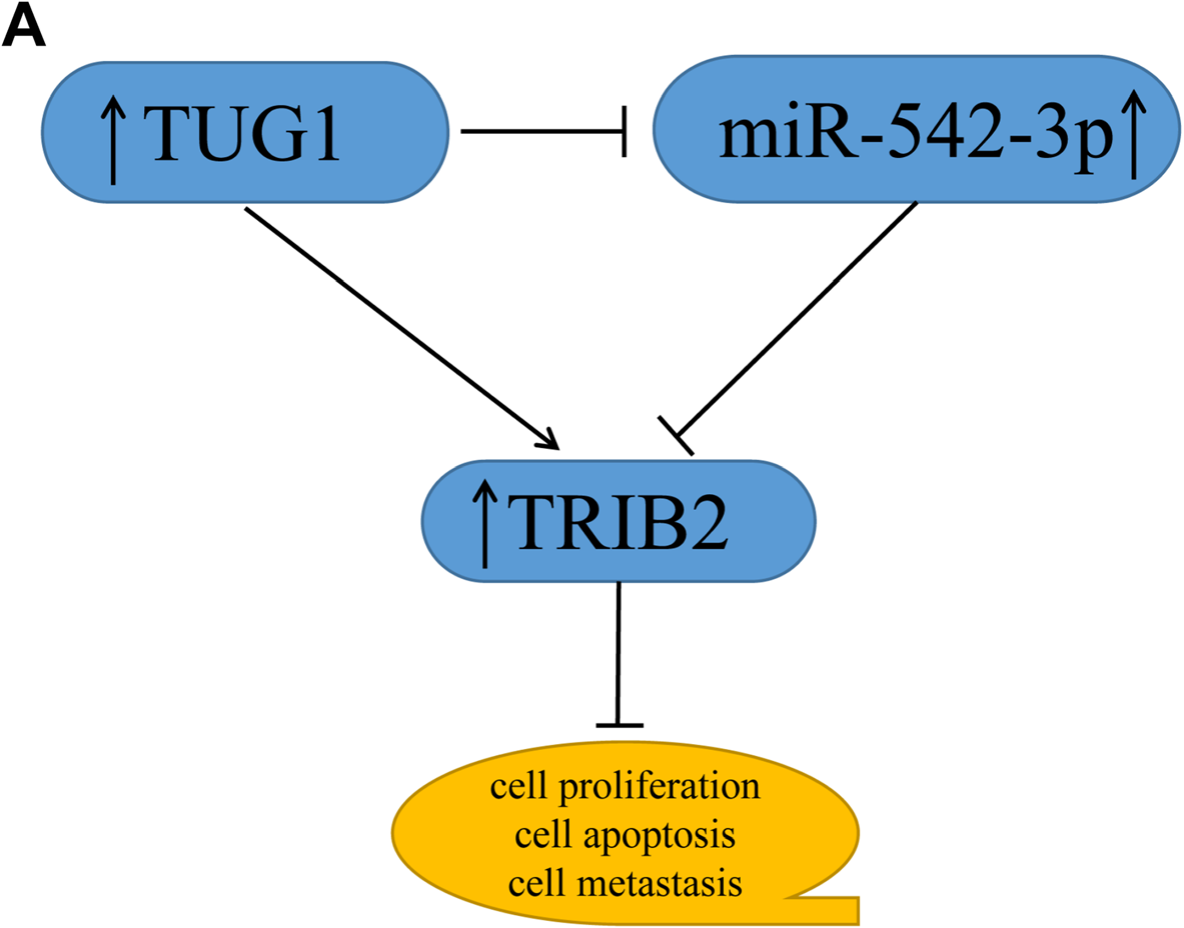
The regulatory role of TUG1/miR-542-3p/TRIB2 axis on CRC cell proliferation, metastasis, and apoptosis

Since lncRNA TUG1 and TRIB2 were both upregulated and their expression was positively associated with each other in CRC tissues, we then transfected CRC cells with si-TUG1 + pc-DNA-TRIB2 or si-TUG1 to explore possible mechanism. The results indicated that TUG1 silencing reduced the mRNA and protein levels of TRIB2 in CRC cells, which could be reversed by up-regulating the expression of TRIB2. Simultaneously, upregulation of TRIB2 could alleviate the impacts of TUG1 silencing-mediated suppression on proliferation, migration, invasion and promotion on apoptosis. Growing evidence has proved the truth that lncRNAs may function as endogenous sponges to interact with miRNAs, thus indirectly modulating genes expression [33, 34]. To investigate the possibility that TUG1 acted as a sponge, bioinformatics analysis was conducted. The result indicated that TUG1 could interact with miR-542-3p and the interaction was verified by the dual-luciferase reporter assay. Subsequently, the level of miR-542-3p in CRC cells transfected with si-TUG1 or pcDNA-TUG1 was measured, and the data confirmed that TUG1 silencing markedly upregulated miR-542-3p, while upregulation of TUG1 significantly downregulated miR-542-3p. Interestingly, we also confirmed that miR-542-3p targeted the 3’UTR of TRIB2. Further studies elucidated that upregulation of miR-542-3p reduced the level of TRIB2, while the effect could be rescued by overexpression of TUG1. Besides, TUG1 depletion inhibited tumor growth and the level of TRIB2 was clearly declined, but the expression of miR-542-3p was markedly increased. To sum up, our results demonstrated that TUG1 silencing impeded the progression of CRC through miR-542-3p/TRIB2 axis (Fig. 8).

 Wnt/β-catenin pathway was involved in diverse cancers [25, 26]. Uppada et al. showed that MASTL induced CRC progression and chemoresistance by promoting Wnt/β-catenin signaling [35]. Qi et al. reported that some Wnt/β-catenin target genes promoted experimental metastasis and migration of CRC cells [36]. In this research, we found that downregulation of TUG1 notably diminished the protein levels of β-catenin, c-Myc, Bcl-2 and upregulated the expression of E-cadherin in CRC cells, whereas downregulation of miR-542-3p or overexpression of TRIB2 reversed the effects. Hence, these results suggested that TUG1 silencing damaged the Wnt/β-catenin pathway through the miR-542-3p/TRIB2 axis, which was in accordance with previous research [23]. The induction action of TUG1 on the Wnt/β-catenin pathway was also confirmed in other cancers, such as osteosarcoma, ovarian cancer and oral squamous cell carcinoma [37–39].

## Conclusions

Our research demonstrated that TUG1 silencing suppressed the progression of CRC by sponging miR-542-3p to reduce TRIB2 expression and the inactivation of Wnt/β-catenin pathway. The TUG1/miR-542-3p/TRIB2 axis might provide potential targets for improving the treatment of CRC.

## Data Availability

The data sets used and/or analyzed during the current study areavailable from the corresponding author on reasonable request.

## Supplementary Infromation

**Additional file 1: Supplementary Figure 1. **TUG1/miR-542-3p/TRIB2 axis regulates the growth of CRC tumor in vivo. At 7 days, 12 days, 17 days, 22 days and 27 days upon injection, 4 mice were euthanized. And the expression of TUG1 (A), miR-542-3p (B) and TRIB2 (C) in xenograft tumor tissues were investigated by qRT-PCR. **P* < 0.05.
