# Supplementary figures and images for: Long noncoding RNA TUG1 regulates the progression of colorectal cancer through miR-542-3p/TRIB2 axis and Wnt/β-catenin pathway
Source: Diagn Pathol. 2021 May 24;16:47. doi: 10.1186/s13000-021-01101-7 (PMC8142490; doi:10.1186/s13000-021-01101-7)

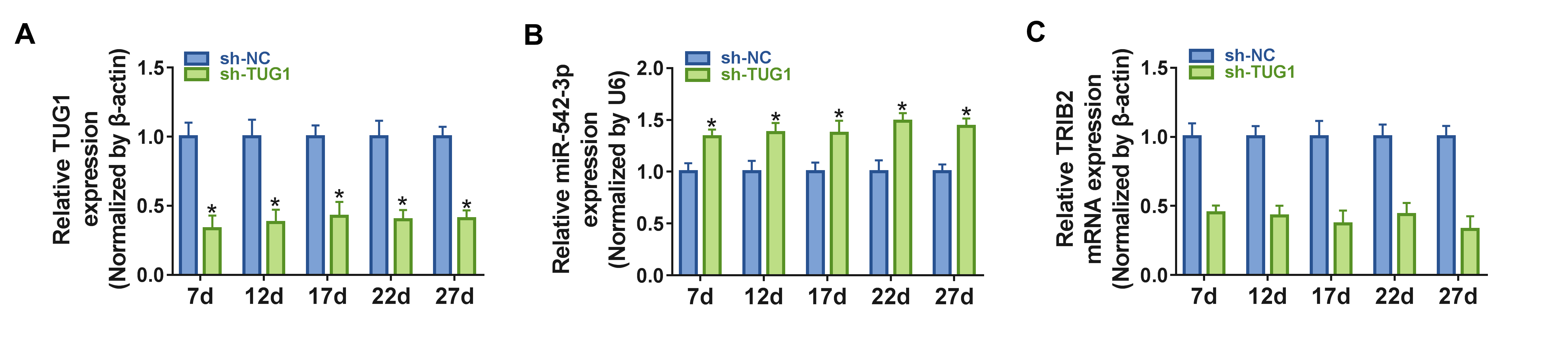

Supplement: Supplementary file 1 — Additional file 1: Supplementary Figure 1. TUG1/miR-542-3p/TRIB2 axis regulates the growth of CRC tumor in vivo. At 7 days, 12 days, 17 days, 22 days and 27 days upon injection, 4 mice were euthanized. And the expression of TUG1 (A), miR-542-3p (B) and TRIB2 (C) in xenograft tumor tissues were investigated by qRT-PCR. *P < 0.05. [file 13000_2021_1101_MOESM1_ESM.tif]
